# Oral Pre-Exposure Prophylaxis by Anti-Retrovirals Raltegravir and Maraviroc Protects against HIV-1 Vaginal Transmission in a Humanized Mouse Model

**DOI:** 10.1371/journal.pone.0015257

**Published:** 2010-12-21

**Authors:** C. Preston Neff, Thomas Ndolo, Apurva Tandon, Yuichiro Habu, Ramesh Akkina

**Affiliations:** Department of Microbiology, Immunology and Pathology, Colorado State University, Fort Collins, Colorado, United States of America; George Mason University, United States of America

## Abstract

Sexual HIV-1 transmission by vaginal route is the most predominant mode of viral transmission, resulting in millions of new infections every year. In the absence of an effective vaccine, there is an urgent need to develop other alternative methods of pre-exposure prophylaxis (PrEP). Many novel drugs that are currently approved for clinical use also show great potential to prevent viral sexual transmission when administered systemically. A small animal model that permits rapid preclinical evaluation of potential candidates for their systemic PrEP efficacy will greatly enhance progress in this area of investigation. We have previously shown that RAG-hu humanized mouse model permits HIV-1 mucosal transmission via both vaginal and rectal routes and displays CD4 T cell loss typical to that seen in the human. Thus far systemic PrEP studies have been primarily limited to RT inhibitors exemplified by tenofovir and emtricitabine. In these proof-of-concept studies we evaluated two new classes of clinically approved drugs with different modes of action namely, an integrase inhibitor raltegravir and a CCR5 inhibitor maraviroc as potential systemically administered chemo-prophylactics. Our results showed that oral administration of either of these drugs fully protects against vaginal HIV-1 challenge in the RAG-hu mouse model. Based on these results both these drugs show great promise for further development as orally administered PrEPs.

## Introduction

With no effective HIV vaccine on the horizon, alternative preventive methods are urgently needed to stem the AIDS epidemic [1]. Although use of condoms can substantially reduce viral transmission, lack of full compliance has been a significant issue [2]. This is especially true in many developing countries where the HIV prevalence is high and condoms are not widely available and/or the compliance is low. Male circumcision has shown reduced viral transmission to men but this does not prevent infection of women [3]. An effective pre-exposure prophylactic (PrEP) that can prevent sexual transmission of HIV-1 is likely to play a major role in preventing millions of new cases [4]. It will also empower women to protect themselves from the HIV risk. The benefits of PrEP in the infectious disease field have been already well documented for the prevention of malaria and mother-to-child transmission in the case of HIV [5], [6], [7]. An effective PrEP, when available, is estimated to prevent 2.7 to 3.2 million new infections in sub-Saharan Africa and thousands of new cases in the high risk individuals in the USA [8].

Currently there are numerous clinically approved effective anti-retroviral drugs that are used to treat the HIV infection and some of these can be potentially exploited for developing an effective PrEP [9], [10]. That PrEP can prevent sexual transmission is substantiated by the early studies in non-human primates which employed daily oral administration of RT inhibitors TDF and/or FTC [11], [12], [13]. This concept has reached clinical trials in which tenofovir is currently being investigated for its prophylactic efficacy [4]. The results of these studies are pending. As can be seen, evaluation of different drug candidates for PrEP has taken a momentum and it is necessary to continually evaluate new candidates for this purpose since a PrEP with proven protective efficacy now may not retain its effectiveness in the future years given the propensity of HIV to develop drug resistance. While the monkey model has been very useful in evaluating appropriate candidate PrEPs, there are a number of limitations for its use to screen large numbers of potential candidates [14], [15]. Chief among these is that it does not use HIV itself for challenge studies in addition to being expensive. This somewhat restricts its predictive value given that many of the present drugs are designed to be specifically effective against HIV, not SIV or SHIV viruses that are used in monkey viral challenges. Furthermore, it is not possible to test candidate PrEPs against genetically divergent and drug resistant viruses that exist in the field.

Humanized mouse models that harbor HIV susceptible human cells and are permissive for HIV infection can overcome these important limitations. In this regard, the classical SCID-hu-PBL humanized mouse model was utilized for early microbicide testing [16], [17], [18]. However, due to low and variable infection rate by vaginal route, it is not considered consistently reliable [19]. Recently there have been substantial improvements in the new generation of humanized mouse models [20], [21], [22]. Transplantation of human hematopoietic stem cells (CD34^+^ cells) into newer generation of immunodeficient mice with much lower innate immunity such as NOD/SCIDγc^−/−^ and Rag2^−/−^γc^−/−^ permitted higher human cell engraftment levels and sustained multilineage human hematopoiesis [20], [21]. Additionally, another model, the BLT mouse model, was developed by a modification of the standard SCID-hu model. This involves transplantation of thymic and liver tissues under the kidney capsule of NOD-SCID mice followed by reconstitution with autologous human CD34^+^ cells [23]. A number of groups including ours have demonstrated the utility of these humanized mice as improved models for HIV-1 infection and CD4 T cell depletion [24], [25], [26], [27], [28], [29]. Furthermore, these models also permit HIV-1 mucosal transmission via both vaginal and rectal routes [30], [31]. Thus it is now possible to experimentally evaluate novel preventive strategies of HIV-1 sexual transmission exploiting these models.

In this context, it was recently shown that tenofovir could prevent HIV-1 vaginal transmission using the BLT mouse model [31]. Using the same model, it was also shown that systemic administration of TDF (tenofovir) and FTC (emtricitabine) prevents HIV-1 infection via vaginal and i/p challenges thus setting the stage for large scale evaluation of different anti-HIV compounds for their efficacy as PrEPs as well as topical microbicides in preventing HIV infection [32]. Using RAG-hu mice, here we evaluated two clinically approved compounds namely, an integrase inhibitor raltegravir and a CCR5 inhibitor maraviroc as a first step to determine their potential as PrEP candidates [10], [33]. Our results show that oral administration of either of these drugs prevents HIV-1 infection via vaginal challenge which is the major route of HIV-1 transmission.

## Materials and Methods

### Generation of humanized Rag2^−/−^γc^−/−^ mice (RAG-hu mice)

Humanized BALB/c-Rag2^−/−^ *γ*c^−/−^ (RAG-hu) mice were prepared by engraftment with human fetal liver-derived CD34^+^ hematopoietic progenitor cells as we previously described [26], [30]. Mice were maintained at the Colorado State University Painter Animal Center. These studies have been reviewed and specifically approved by the CSU Institutional Animal Care and Use Committee (Protocol 09-1460A). Briefly, newborn mice were conditioned by irradiating with 350 rads and then injected intrahepatically with 0.5-1×10^6^ human CD34^+^ cells. Mice were screened for human cell engraftment at 10–12 weeks post-reconstitution. Peripheral blood was collected by tail bleed and red blood cells were lysed by using the Whole Blood Erythrocyte Lysing Kit (R&D Systems, Minneapolis, MN). The white blood cell fraction was stained with antibodies against the human pan-leukocyte marker CD45 (Caltag) and FACS analyzed to determine the levels of human cell engraftment as we previously described [26]. To assure efficient infection, mice with over 40% human cell engraftment (as listed in Table 1) were chosen for vaginal viral challenges.

**Table 1:** Summary of human cell engraftment levels in humanized (RAG-hu) mice*.

Uninfected Control			Non-Treated		
Mouse	Gender	%Engraftment	Mouse	Gender	%Engraftment
J667	Female	95	812	Female	91.6
J666	Female	70	811	Female	69.3
			810	Female	91.4
			809	Female	83.9
			J635	Female	45.4
			J634	Female	83.2
			J632	Female	75

Raltegravir Treated			Maraviroc Treated		
Mouse	Gender	%Engraftment	Mouse	Gender	%Engraftment
J683	Female	43.6	J672	Female	74.3
J682	Female	47	J671	Female	65.8
J681	Female	60.5	J670	Female	80.4
J680	Female	62.6	J642	Female	81.1
J637	Female	83.5	J641	Female	62.4
J636	Female	70	J640	Female	67.2

*Peripheral blood was collected from human CD34 cell reconstituted mice at 10–12 weeks post engraftment. White blood cell fraction was stained with human CD45 FITC conjugated antibody and analyzed by FACS to confirm human cell engraftment prior to drug treatments and vaginal HIV challenges.

### Oral administration of anti-HIV drugs raltegravir and maraviroc and HIV-1 challenge by vaginal route

Female RAG-hu mice were administered with either raltegravir or maraviroc by oral gavage (6 mice each). Clinical formulations of these drugs in tablet form (Maraviroc (Selzentry) 150 mg, Pfizer Labs; Raltergravir (Isentress) 400 mg, Merck & Co) were freshly dissolved in distilled water each day prior to oral gavage. Mouse equivalent drug doses were calculated by using an interspecies allometric scaling factor of 12.3 to arrive at 164 mg/kg and 62 mg/kg doses for raltegravir and maraviroc respectively [34], [35]. Mice (six per group) received either raltegravir (3.28 mg per 20 gram mouse) or maraviroc (1.23 mg per 20 gram mouse) by oral gavage daily. Mice were challenged with HIV-1 vaginally on the 4^th^ day of treatment and the drug treatment continued for 3 more days. For vaginal viral challenges, cell-free HIV-1 strain BaL-1 (R5 tropic virus) contained in the original media used to produce the virus (RPMI 1640 medium supplemented with 10% fetal bovine serum) was used. Vaginal infections were performed in a volume of 20 µl (3000 TCID of BaL-1 virus). Sterile P200 tips that had been previously heated over a flame to smooth any abrasive surfaces were used to deliver the virus [30]. Anesthetized mice were held in an inverted position for four minutes post-inoculation to allow virus to adsorb and to prevent immediate discharge of virus. Control non-treated mice (n = 7) were also challenged similarly by the vaginal route. Mice were observed daily and blood samples drawn weekly to assess plasma viremia.

### Measurement of viral loads

To detect HIV-1 in plasma of infected mice by Q-RT-PCR, RNA was extracted from 25–50 µl of EDTA-treated plasma using the QIAamp Viral RNA kit (Qiagen, Valencia, CA). Q-PCR was performed using a primer set specific for the HIV-1 LTR sequence and a corresponding LTR specific probe as described previously [30], [36]. To detect integrated virus, cellular DNA was extracted using Qiagen kit. The cellular DNA was subjected to Q-PCR to determine the proviral loads.

### Flow cytometry

Whole blood was collected and red blood cells lysed as reported previously [26], [36]. Peripheral blood cells were stained for hCD3-PE and hCD4-PECy5 (Caltag) markers and analyzed using a Coulter EPICS XL-MCL FACS analyzer (Beckman Coulter, Fullerton, CA). CD4^+^ T cell levels were calculated as a ratio of the entire CD3 population (CD4^+^CD3^+^:CD4^−^CD3^+^). To establish baseline CD4^+^ T cell ratios, all mice were analyzed prior to infection.

## Results

### Oral administration of integrase inhibitor raltegravir protects humanized mice from HIV-1 infection via vaginal challenge

We have previously shown that RAG-hu mice are susceptible to HIV-1 infection via both vaginal and rectal routes [30]. Here we used this model to determine if systemic administration of raltegravir protects against vaginal HIV-1 challenge.

**Figure 1:**
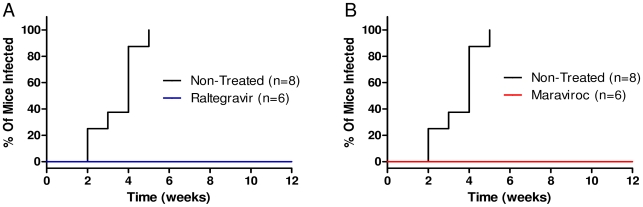
Oral administration of raltegravir or maraviroc protects humanized mice against vaginal HIV-1 challenge. RAG-hu mice were challenged by vaginal route after oral administration of raltegravir or maraviroc as described in Methods. Blood was collected weekly from infected mice and the status of HIV-1 infection was determined by Q-RT-PCR. The viral challenge experiments were performed at same time for both of the drugs and the same set of control non-treated infected mice were used for comparison. Kaplan-Meier plots of time course of appearance of viremia in drug treated versus non-treated virus challenged mice. A. Raltegravir treated B. Maraviroc treated.

**Figure 2:**
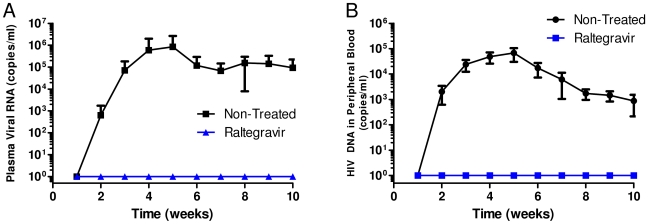
RNA and DNA viral loads in mice administered with raltegravir. RAG-hu mice were challenged by vaginal route after oral administration of raltegravir as described in Methods. Blood was collected weekly. Viral RNA was extracted from the plasma fraction and DNA was extracted from the cellular fraction. Viral RNA and DNA loads were determined by Q-RT-PCR as described in methods. A. RNA viral loads B. DNA viral loads.

Mice were administered with the drug daily by oral gavage since this drug is taken orally in a clinical setting. Vaginal viral challenge was performed on the 4th day and the drug treatment continued for three more days. To determine the status of HIV infection, mouse plasma and cellular blood fractions were analyzed by Q-PCR on a weekly basis. Our results showed that all of the non-treated infected mice became virus positive by the 5th week post challenge (Fig. 1A). Persistent viremia in plasma and proviral loads in the cellular fractions were observed throughout the evaluation period with viral loads reaching up to 10^6^ copies/ml (Fig. 2). In contrast, none of the raltegravir treated mice became infected at 5 weeks post-viral challenge unlike the non-treated mice (Fig. 1A). Since it is possible that the drug treatment might have delayed the onset of infection, mice were followed for an additional 5 weeks. No evidence of infection was seen throughout the 10 week observation period as evaluated by either RNA or DNA PCR (Fig. 2). These data collectively suggest that oral administration of raltegravir fully protects mice against HIV-1 viral challenge. With regard to any adverse effects such as loss of appetite or weight loss, none was noted during the entire experimental period and the mice appeared normal.

### Oral administration of CCR5 inhibitor maraviroc protects humanized mice from HIV-1 infection via vaginal challenge

**Figure 3:**
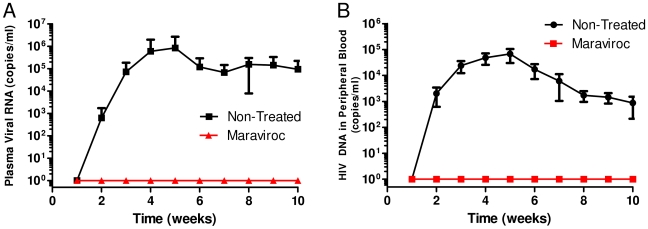
RNA and DNA viral loads in mice administered with maraviroc. RAG-hu mice were challenged by vaginal route after oral administration of raltegravir as described in Methods. Blood was collected weekly. Viral RNA was extracted from the plasma fraction and DNA was extracted from the cellular fraction. Viral RNA and DNA loads were determined by Q-RT-PCR as described in methods. A. RNA viral loads B. DNA viral loads.

In addition to the viral integrase inhibitor raltegravir, we also evaluated a CCR5 antagonist maraviroc to determine its efficacy in preventing HIV-1 infection via vaginal challenge using a similar protocol like above. This experiment was done at the same time and the same non-treated virus infected animals were used as controls. Maraviroc was also administered orally like above in a similar time scale. Our results showed that while all the seven control untreated mice became virus positive by the fifth week, none of the six maraviroc treated mice became infected throughout the ten week observation period (Fig. 1B). Both RNA PCR to detect plasma viremia and DNA PCR to detect integrated provirus in blood cellular fractions were negative in maraviroc treated mice in contrast to non-treated virus challenged mice (Fig. 3). These results showed that oral administration of maraviroc fully protects humanized mice against vaginal infection.

### CD4 T cell loss in non-drug treated mice versus raltegravir and maraviroc treated mice following vaginal infection

**Figure 4:**
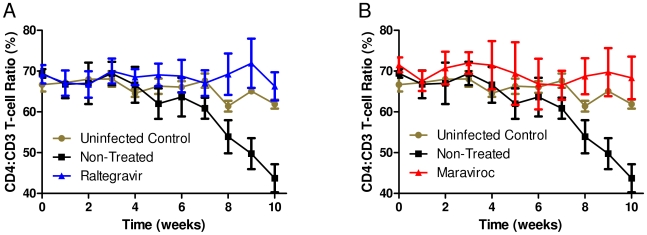
CD4 T cell decline in non-treated vaginally challenged mice in contrast to mice protected with raltegravir and maraviroc treatment. Levels of CD4 T cells were monitored on a weekly basis by FACS to determine their decline in treated versus non-treated mice. Baseline values for each of the mice were established prior to infection as described in Methods. A. Raltegravir treated, B. Maraviroc treated.

The above criteria of viral detection showed that both raltegravir and maraviroc treated mice were fully protected from vaginal HIV-1 challenge. Since CD4 T cell loss is a main characteristic of HIV-1 infection in humanized mice akin to that seen in the human, we further evaluated the virus challenged mice for any evidence of such loss [26], [36]. Accordingly, peripheral blood was collected weekly and subjected to FACS analysis. Baseline CD4 T cell levels for each of the experimental mice were determined prior to viral challenge and these values were compared to the levels post-viral challenge. While there was a clear pattern of CD4 T cell decline in un-treated mice, their levels were stable in both groups of mice receiving raltegravir or maraviroc further confirming the absence of HIV-1 infection in these mice (Fig. 4).

## Discussion

Here we have shown that oral administration of two clinically approved drugs namely, raltegravir or maraviroc fully protect humanized mice against HIV-1 infection from vaginal viral challenge suggesting their potential utility as PrEPs. These two compounds have different modes of action [10]. Maraviroc is a low molecular weight CCR5 antagonist which inhibits the binding of the natural ligands of CCR5, namely chemokine ligand 3 (CCL3 also known as MIP-1a), CCL4 (MIP1-b) and CCL5 (RANTES) [37]. It is a functional CCR5 antagonist devoid of agonist activity and shown to have a long lasting physical and functional occupancy of CCR5 leading to sustained antiviral activity [38]. It has been shown to have potent effect against all R5 tropic viruses representing various viral clades in addition to being effective against a wide range of drug resistant viruses [39]. Topical vaginal application of maraviroc as a microbicide was recently shown to protect rhesus monkeys against SHIV virus challenge [40]. Raltegravir is an integrase strand transfer inhibitor that interferes with the viral DNA integration which is an essential step in viral replication. It is active against multidrug-resistant and both CCR5-tropic and CXCR4-tropic HIV-1 strains [33], [41]. To our knowledge this is the first report evaluating these two drugs as potential systemic PrEPs against HIV-1 vaginal transmission.

To simulate the clinical situation in mice, we administered each of the drugs orally as the prescription suggested for human use thus permitting intestinal absorption and reaching systemic effective concentrations. With regards to oral dosing in these proof-of-concept studies, we treated the animals for three days with the drug to achieve a systemic drug equilibrium in vivo prior to vaginal challenge and continued the drug treatment for an additional four days. This is similar to the studies of Denton et al in BLT mice that employed FTC/TDF (Truvada) for PrEP testing [32]. Whereas the drug combination FTC/TDF (Truvada) was injected i/p to the mice to demonstrate PrEP efficacy in the above studies, we used oral administration as clinically suggested for the above drugs. While all the control non-treated, vaginally HIV-1 challenged mice became infected within five weeks, none of the raltegravir or maraviroc treated mice (6 mice each) showed any evidence of infection. Furthermore, DNA extracted from splenic tissue samples after euthanizing the mice at sixteen weeks post-challenge also did not show any evidence of infection by PCR analysis (data not shown). Thus protection conferred by either of these two drugs is highly significant (p value 0.0006, Fisher's exact test). Since both maraviroc and raltegravir treatments fully protected against vaginal challenge, this also confirmed that effective protective concentrations for both these drugs were reached and maintained in the vaginal tissues during the oral dosing period. We further evaluated the mice for evidence of helper CD4 T cell loss which is a characteristic hallmark of HIV-1 infection. As expected, a declining trend for CD4 T cell counts was observed in control non-treated mice in contrast to either of the treatment groups receiving raltegravir or maraviroc (Fig. 4). These data collectively showed that treated mice resisted vaginal viral challenge thus indicating full protection in contrast to non-treated mice.

Whereas topical microbicides received the major attention other than vaccines to preventing HIV infection thus far with many clinical trials currently ongoing in the field, experimental studies on systemic PrEPs for HIV have been limited to very few compounds with a main focus on RT inhibitors [13], [42], [43]. These included tenofovir, emtricitabine and efavirenz which showed efficacy in non-human primates against i/v, vaginal or rectal challenges with either SIV and/or different versions of SIV/HIV chimeric viruses [13], [42]. In addition to showing efficacy in the monkey models, the RT inhibitors tenofovir and emtricitabine also showed efficacy in the BLT mouse model against HIV challenge [32]. Based on the effectiveness of tenofovir as a PrEP in the experimental studies it is currently in clinical trials to evaluate its efficacy in the human [44]. With regard to fusion inhibitors, oral administration of CMPD167, a small molecule CCR5 inhibitor, protected macaques against vaginal SHIV viral challenge [45]. As can be seen, there is a paucity of number of compounds tested for systemic PrEP.

Our present results have provided the proof-of-concept data for further investigating the potential of raltegravir and maraviroc as PrEPs thus identifying additional novel class of molecules with different modes of action [4], [10]. Based on their proven broad spectrum of activity against divergent HIV strains in the clinic, both these drugs make excellent candidates for PrEP. Future studies should evaluate variations in the dose, timings of drug administration prior to vaginal challenge and duration of efficacy without further dosing after viral challenge to determine the memory effect. It is also necessary that field and drug resistant viruses be tested in this humanized mouse model. Furthermore, use of more than one drug in any PrEP will be more effective in field conditions. This can also be tested in this mouse model using a combination of raltegravir and maraviroc to derive pre-clinical data. Such evaluations will fine tune the PrEP regimens to be more practically applicable for clinical testing.

In addition to the systemic PrEP, another highly promising method of prevention of HIV-1 sexual transmission is the topical use of effective microbicides as mentioned above. Therefore, testing of raltegravir and maraviroc as topical microbicides in the RAG-hu mouse model of sexual HIV-1 transmission is likely to provide critical pre-clinical data in this context as well.
